# Time‐dependent changes in cardiovascular function during copulatory behavior induced by the hand method in the male dog

**DOI:** 10.1007/s12522-013-0165-x

**Published:** 2013-07-27

**Authors:** Misao Terada, Yatsuka Horii, Fumio Sato, Kazumi Taniguchi, Tatsuya Hori, Eiichi Kawakami, Toshihiko Tsutsui, Toshio Akimoto, Motoo Shinoda, Toru R. Saito

**Affiliations:** ^1^ Laboratory Animal Research Center Dokkyo Medical University 880 Kitakobayashi 321‐0293 Mibu Tochigi Japan; ^2^ Kitayama Labes Co., Ltd. Nagano Japan; ^3^ Japan Racing Association, Hidaka Training and Research Center Hokkaido Japan; ^4^ Department of Veterinary Anatomy Kitasato University Aomori Japan; ^5^ Department of Reproduction Nippon Veterinary and Life Science University Tokyo Japan; ^6^ Division of Laboratory Animal Science Nippon Medical School Tokyo Japan; ^7^ Behavioral Neuroscience Laboratory Nippon Veterinary and Life Science University Tokyo Japan

**Keywords:** Adrenaline, Hand method, Heart rate, Male dog, Noradrenaline

## Abstract

**Purpose:**

Ejaculation in the male dog consists of three fractions. Observation of behavior and measurement of heart rate (HR), and plasma noradrenaline (NA) and adrenaline (Ad) concentrations were researched sequentially, and a fundamental examination of the features of sympathetic nerve activity during copulatory behavior induced by the hand method in the male dog was undertaken.

**Methods:**

We investigated the breeding capability of male dogs. HR, plasma NA level and plasma Ad levels were measured during ejaculation induced by the hand method.

**Results:**

HR was 125.8 ± 6.0 beats/min at rest, and peaked during mounting at 195.2 ± 8.2 beats/min. Moreover, HR at 3 min after the first fraction decreased to values similar to those at rest. Plasma NA and Ad concentrations during copulatory behavior induced by the hand method did not differ significantly from those at rest. However, although there was no significant difference, plasma NA concentration during ejaculation of the third fraction peaked at about 1.8 times the baseline value.

**Conclusions:**

In the male dog, excitation of sympathetic nerves of long duration during erection of the penis and ejaculation is questionable. However, inhibition of sympathetic nerves and activation of parasympathetic nerves is thought to occur during erection of the penis and ejaculation.

## Introduction

Previous reports of sudden cardiac death (SCD) after ejaculation have suggested an important relationship between male sexual behavior and the circulatory system [1, 2, 3]. Ejaculation is the most important reproductive function of the mammalian male, and understanding the underlying mechanisms may help improve reproductive technologies and the treatment of reproductive disorders. In addition, elucidating the relationship between ejaculation and cardiac function might contribute to safer reproductive behaviors.

Basic sexual behavior data are essential because patterns of sexual behavior vary with species. We have previously reported a change in heart rate (HR) in stallions and both young adult and aged male rats during copulation, as measured using a telemetry system, showing the function of the autonomic nervous system [4, 5, 6, 7] and indicating that copulatory behavior increases the load on the circulatory system. However, in‐depth research on physiological indices in the male dog during copulatory behavior has yet to be conducted. Moreover, no studies have monitored continual changes in HR and sympathetic nerve activity during copulatory behavior. The male dog performs ejaculation in three fractions. During ejaculation of these three fractions, the *Bulbus glandis* in the male dog (also called the ‘tie’ or ‘lock’) becomes dilated and connects the male dog with the female dog. This behavior continues for about 5–30 min [8]. Judging the timing of each fraction is, therefore, difficult, because the male penis has been inserted in the female vagina during ejaculation. In this study, behaviors, HR, and plasma noradrenaline (NA) and adrenaline (Ad) concentrations were determined sequentially, and a fundamental examination of the features of sympathetic nerve activity during copulatory behavior induced by the hand method in male dogs was undertaken.

## Materials and methods

### Animals

Male beagle dogs aged 2–8 years (*n* = 6) and 1‐year‐old female dogs (*n* = 2) were obtained from the Imamichi Institute for Animal Reproduction (Ibaraki, Japan) and the Department of Reproduction, Nippon Veterinary and Life Science University (Tokyo, Japan). The animals were acclimatized in the animal room, with twice‐daily feeding and free access to water, for at least 1 month before the experiments.

### Observation of hand‐induced sexual behavior and measurement of HR

**Figure 1:**
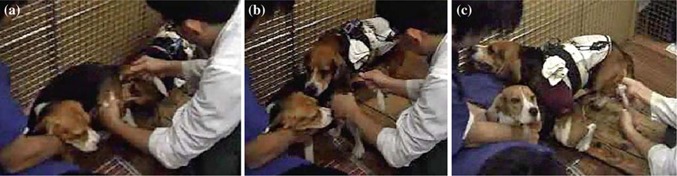
Copulatory behavior by the hand method in the male dog. **a** Exposing female dog to male dog and sniffing; **b** Mounting; **c** Ejaculation by the hand method

All observations of behavior were conducted using a video camera and an observer. First, a male dog with a telemetry system was placed in the observation cage (120‐cm D × 200‐cm W × 120‐cm H). Fifteen minutes later, a sexually receptive female dog was introduced into the same cage. The male dog then mounted the female and the hand of a researcher was used to induce copulatory behavior in the male dog. After ejaculation of the third fraction, the dogs were separated (Fig. 1).

**Figure 2:**
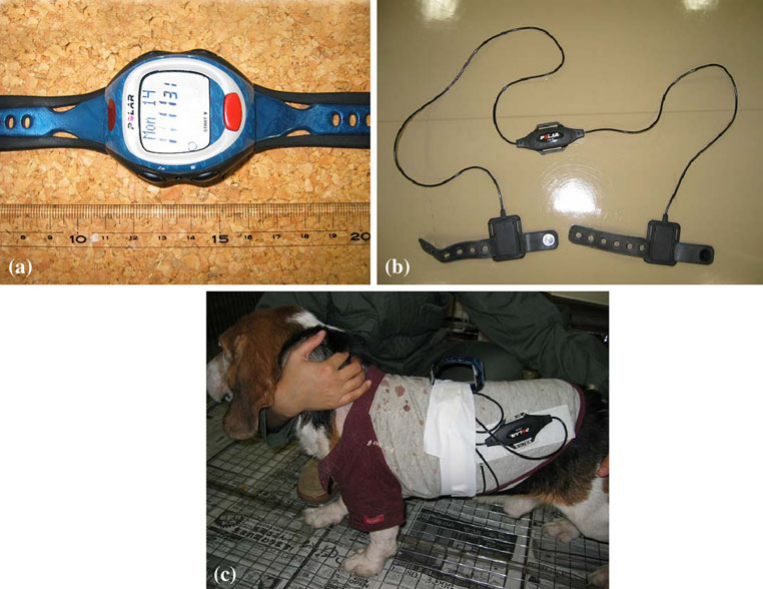
Method for measuring heart rate by telemetry **a** Receiver; **b** Transmitter; **c** Measurement of heart rate

Measurement of HR was performed using a telemetry system. A T51H transmitter (Polar Electro Oy, Kempele, Finland) was attached to the chest of the male dog and transmitted data were recorded on an S610 receiver (Polar Electro Oy) (Fig. 2). HR measured within the observation cage was considered to be resting HR, and was recorded at 5‐s intervals until 60 min after the first ejaculation fraction. Change in HR was measured at the following 14 time points: rest; exposure of the female dog to the male dog; mounting; first ejaculation fraction; second ejaculation fraction; third ejaculation fraction; and 1, 2, 3, 4, 5, 10, 30 and 60 min after the first fraction. Values for each time point were determined as the mean of three measurements taken at the time the animal showed the particular behavior and 5 s before and after.

### Blood sampling and measurement of plasma NA and Ad concentrations

Blood samples (2 ml) were collected into tubes containing heparin sodium (VENOJECT; Terumo, Tokyo, Japan) via the right cephalic vein with an indwelling needle and catheter at the following 10 time points: rest; exposure of the female dog to the male dog; mounting; first ejaculation fraction; second ejaculation fraction; third ejaculation fraction; and 5, 10, 30 and 60 min after the first fraction. All blood samples were kept on ice until centrifugation at 2,200×*g* for 15 min at 4 °C, and plasma fractions were stored at −80 °C until analysis. Plasma NA and Ad concentrations were measured by high‐performance liquid chromatography (C‐R7A; Shimadzu, Tokyo, Japan) using a system equipped with an electrochemical detector (Nanospace SI‐1 2005; Shiseido, Tokyo, Japan), as described previously [5, 6, 9].

### Statistical analysis

All data are presented as mean ± standard error of the mean. Statistical analysis was performed by two‐way repeated analysis of variance followed by Dunnett's test. Values of *P* < 0.05 were considered to represent a significant difference.

## Results

### Heart rate

**Figure 3:**
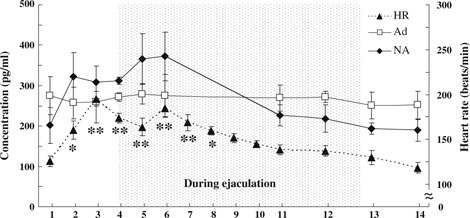
Change in heart rate and plasma adrenaline and noradrenaline concentrations during copulatory behavior by the hand method in the male dog. *1* Rest time; *2* Exposing female dog to male dog; *3* Mounting; *4* First fraction; *5* Second fraction; *6* Third fraction; *7* 1 min after first fraction; *8* 2 min after first fraction; *9* 3 min after first fraction; *10* 4 min after first fraction; *11* 5 min after first fraction; *12* 10 min after first fraction; *13* 30 min after first fraction; *14* 60 min after first fraction. All data are presented as mean ± S.E.M. Dunnett's test: * *P* < 0.05 vs. each rest time; ** *P* < 0.01 vs. each rest time

Continual changes in HR and plasma Ad and NA concentrations during copulatory behavior induced by the hand method in male dogs are shown in Fig. 3. At rest, HR was 125.8 ± 6.0 beats/min. On exposing the female dog to the male dog, HR increased to 160.7 ± 10.3 beats/min, significantly higher than at rest (*P* < 0.05). HR peaked at 195.2 ± 8.2 beats/min during mounting. HRs at ejaculation of the first and second fractions were 174.0 ± 5.4 and 164.0 ± 9.5 beats/min, respectively, lower than peak HR. However, HR at ejaculation of the third fraction increased to 185.1 ± 10.3 beats/min, higher than HRs at ejaculation of the first and second fractions. From mounting to ejaculation of the third fraction, HR was significantly higher compared to that at rest (*P* < 0.01). After ejaculation of the third fraction, HRs at 1 and 2 min after ejaculation of the first fraction were 168.7 ± 8.7 and 159.6 ± 4.8 beats/min, respectively (*P* < 0.01 and *P* < 0.05, respectively). HR decreased after ejaculation of the third fraction and HR at 3 min after first fraction was not significantly different from that at rest. Therefore, HR decreased to a level similar to that at rest within 2 min.

### Plasma NA and Ad concentrations

Ad concentrations at rest, exposure of the female dog to the male dog, at mounting and at ejaculation of the first fraction were 275.0 ± 46.4, 257.0 ± 38.4, 259.4 ± 52.8 and 271.9 ± 11.0 pg/ml, respectively. Ad peaked at 279.5 ± 25.3 pg/ml at ejaculation of the second fraction, but no significant difference from the baseline value at rest was evident. However, Ad concentrations at 5, 10, 30 and 60 min after ejaculation of the first fraction were 269.4 ± 32.7, 271.9 ± 14.6, 251.3 ± 32.8 and 252.0 ± 34.2 pg/ml, respectively, and did not differ significantly from the value at rest.

NA concentration at rest was 201.3 ± 44.2 pg/ml. NA concentrations at exposure of the female dog to the male dog, at mounting, and at ejaculation of the first and second fractions were 322.6 ± 59.6, 308.5 ± 40.8, 312.0 ± 8.8 and 366.2 ± 62.9 pg/ml, respectively; each value represents a trend toward an increase. NA concentration rose to about 1.8‐fold that at rest, and peaked at 372.2 ± 59.8 pg/ml at ejaculation of the third fraction. However, no significant difference from the value at rest was seen. NA concentrations at 5, 10, 30 and 60 min after ejaculation of the first fraction were 227.0 ± 26.1, 218.1 ± 33.8, 193.2 ± 14.6 and 189.7 ± 26.9 pg/ml, respectively, and did not differ significantly from the value at rest.

### Analysis of copulatory behavior

Ejaculation times for the various fractions, as induced by the hand method, and the behavior of male dogs during copulatory behavior are shown in Table 1. Ejaculation times for the first, second and third fractions induced by the hand method were 11.3 ± 1.3, 29.0 ± 3.6 and 1,197.7 ± 118.2 s. Moreover, the time from first fraction to contraction of the penis by the hand method was 1,235.7 ± 119.7 s.

**Table 1:** Ejaculation time of various fraction in the male dog

	First fraction (seconds)	Second fraction (seconds)	Third fraction (seconds)	From first fraction to contraction of a penis (seconds)
Mean ± SEM	11.3 ± 1.3	29.0 ± 3.6	1,197.7 ± 118.2	1,235.7 ± 119.7

## Discussion

In the present study, changes in HR, plasma Ad level and plasma NA level were monitored continuously during copulatory behavior induced by the hand method in male dogs. HR peaked at the time of mounting (Fig. 2). This result differs from findings in rats and humans, in which HR peaked at ejaculation and orgasm, respectively [4, 10]. However, this result is similar to the peak HR occurring during copulatory behavior in stallions [6]. The influence of exercise on supporting the body on the hind legs during mounting is a plausible cause of this. Moreover, mental stress and difficulty of emotional control peculiar to the dog may also be involved [11]. During copulatory behavior in humans and male rats, HR peaked at the time of ejaculation [4, 10]. Time of ejaculation, duration of contact with the female, and amount of seminal fluid are short/small in humans and male rats. On the other hand, the volumes of seminal fluid in the first, second and third fractions in dogs are 0.1–3.0, 0.5–4.0 and 1–30 ml, respectively [12, 13]. Moreover, because the duration of ejaculation is long, so is the duration of contact with the male dog. In a male dog that shows this copulatory behavior pattern, HR during ejaculation is thought to fluctuate easily according to psychological and physical influences.

The times to ejaculation of the first and second fractions were short, and the third fraction began to be ejaculated within 1 min after the first fraction had become clear. HR decreased to a level similar to that at rest within 2 min. HR thus decreased to a level similar to that at rest during ejaculation of the third fraction, implying that sexual arousal decreased at this time.

The changes in plasma Ad concentration during copulatory behavior induced by the hand method in male dogs resembled those in HR. In contrast, plasma NA concentration increased beginning with exposure of the female dog to the male dog, peaked during ejaculation of the third fraction, and tended to decrease thereafter. Kruger et al. [14, 15] reported that temporary activation of the sympathetic nervous system during sexual arousal and orgasm in humans is caused by changes in Ad and NA levels during masturbation. This is similar to the findings of Kruger et al., and the sympathetic nerve is thought to react in a dominant way during copulatory behavior induced by the hand method in the male dog, playing a critical role. Wiedeking et al. [16] reported that erection of the penis and potency of sexual arousal in humans correlate strongly with the plasma NA concentration. Thus, when the penis is fully erect and sexual arousal is high, the plasma NA concentration is high, and the sympathetic nervous system is greatly reactive. However, plasma NA concentration decreased to the baseline resting level by 5 min after ejaculation of the first fraction in male dogs, which is indicative of sustained erection of the penis and ejaculation. Moreover, HR has been reported to change in an identical manner, and erection of the penis in the male dog is thought to be maintained via a spinal cord reflex [17, 18]. In the male dog, excitation of the sympathetic nerves for a long duration during erection of the penis and ejaculation appears questionable. However, inhibition of the sympathetic nervous system and activation of the parasympathetic nervous system is thought to occur during both erection of the penis and ejaculation.

We studied the relationship between the autonomic nervous system and circulatory system during copulatory behavior by measuring HR and plasma catecholamine concentrations in copulating male dogs using the hand method. Strong correlations have been seen between HR, plasma catecholamine concentrations, and blood pressure (BP) [19, 20]. BP is affected by changes in HR and stroke volume, arterial extensibility, ejection speed and peripheral resistance [21]. Systolic BP (SBP), in particular, is affected by changes in stroke volume, arterial extensibility, and ejection speed, whereas diastolic BP is affected by changes in HR, SBP, arterial extensibility, and peripheral resistance induced through the actions of catecholamine. Our previous study reported on HR, plasma catecholamine concentration and BP during copulatory behavior in male rats [5]. Those findings suggested that stroke volume decreases suddenly with HR just after ejaculation, resulting in a sudden decrease in SBP. BP, and SBP in particular, was thus suggested to play a role in immediate reductions to rapid circulatory organ load. Given the present results, the same role of BP is suggested in male dogs.

In humans, coronary artery disease and myocardial abnormality including fibromuscular dysplasia of the atrioventricular nodal artery have been reported as causes of SCD after ejaculation [2]. Moreover, in thoroughbred stallions, myocardial findings such as hemorrhage, swelling, fibrosis and infarction and aortic root rapture have been reported as causes of SCD after ejaculation [22]. However, no reports have described SCD after ejaculation in male dogs. A number of factors may contribute to the lack of reports describing SCD after ejaculation in male dogs. In this study, HR after ejaculation decreased to a level similar to that at rest in the early stages. This result represents an early decrease in HR, even if compared with humans or thoroughbred stallions. The possibility that the cardiac load on the heart in male dogs with heart disease is reduced by early decreases in HR is suggested.

By undertaking a comparison of humans and thoroughbred stallions with reports of SCD after ejaculation with the situation in male dogs and male rats without such reports, we hope to contribute to clarification of the underlying mechanisms and their prevention.

## Acknowledgments

The author thanks Dr. Nagy GM., Neuroendocrine Research Laboratory, Department of Human Morphology, Hungarian Academy of Science and Semmelweis University. We also thank all the staff in his laboratory.

### Conflict of interest

We have no conflict of interest.
